# Institutional Returns for 1919

**Published:** 1920-04-24

**Authors:** 


### INSTITUTIONAL RETURNS FOR 1919.

Aberdeen Royal Asylum.—The report again appears in a restricted form, owing to "the continual abnormal cost of printing." 1,129 patients were treated during the year; of 146 discharges 78 had recovered; mental disturbance in 14 cases was attributed to influenza, 11 to syphilis, and 22 to alcoholic excess.

Brighton County Borough Asylum, Haywards Heath.—The maintenance rate of patients now stands at 20s. a week. The death-rate for male patients has been exceptionally high, chiefly on account of the large preponderance of old and debilitated patients amongst admissions, but overcrowding and lower vitality as the result of malnutrition have contributed to this result. The female rate has fallen to the pre-war standard.

Glamorgan County Lunatic Asylum.—The county maintenance rate increased from 12s. 10d. per week in the first quarter to 14s. in the last quarter. There was a daily average residence throughout 1919 of 1,771. Of 182 discharges 98 were recoveries. There were seven inquests in respect of accidental deaths.

Monmouthshire Asylum.—The medical superintendent is glad to report that by the end of the year under review the nutrition of the patients generally was improving again. There was an epidemic of typhoid fever in the institution: nineteen patients were attacked and six died.

Inverness District Asylum.—The average daily residence of patients in 1919 was 706. Among the predisposing causes hereditary or family predisposition to insanity was found to exist in 58 cases. The asylum won a first prize at Inverness Agricultural Show for potatoes.

The Retreat, Yorkshire.—Twenty-one of thirty-nine male nurses were with the armies, and one men's department was taken over entirely by women, and maids undertook most of the ward work. Of the cases, all of mental diseases, two were persons who had taken active part in occult or spiritualistic practices.

London Missionary Society.—The year's finances are made up to March 31. The Society's work extends to all parts of the world, and the annual report, illustrated by many maps, is full of interest. The total income for the year was £326,519.

King Edward VII. Sanatorium, Midhurst.—The report for the year ending July 1919 shows that of 301 cases 69 had the disease arrested, 189 were improved, and 43 were stationary or worse. The cost of the institution is not dealt with in the report.

